# Exploring the Future of Edible Insects in Europe

**DOI:** 10.3390/foods11030455

**Published:** 2022-02-03

**Authors:** Simone Mancini, Giovanni Sogari, Salomon Espinosa Diaz, Davide Menozzi, Gisella Paci, Roberta Moruzzo

**Affiliations:** 1 Department of Veterinary Sciences, University of Pisa, Viale delle Piagge 2, 56124 Pisa, Italy; simone.mancini@unipi.it (S.M.); salomon.espinosadiaz@phd.unipi.it (S.E.D.); gisella.paci@unipi.it (G.P.); roberta.moruzzo@unipi.it (R.M.); 2 Interdepartmental Research Center “Nutraceuticals and Food for Health”, University of Pisa, Via del Borghetto 80, 56124 Pisa, Italy; 3 Department of Food and Drug, University of Parma, Parco Area delle Scienze 27/A, 43124 Parma, Italy; davide.menozzi@unipr.it

**Keywords:** entomophagy, novel food, mealworm, grasshopper, cricket, locust, acceptance, alternative protein, sustainability, neophobia

## Abstract

The effects of population increase and food production on the environment have prompted various international organizations to focus on the future potential for more environmentally friendly and alternative protein products. One of those alternatives might be edible insects. Entomophagy, the practice of eating insects by humans, is common in some places but has traditionally been shunned in others, such as European countries. The last decade has seen a growing interest from the public and private sectors to the research in the sphere of edible insects, as well as significant steps forward from the legislative perspective. In the EU, edible insects are considered novel foods, therefore a specific request and procedure must be followed to place them in the market; in fact, until now, four requests regarding insects as a novel food have been approved. Insects could also be used as feed for livestock, helping to increase food production without burdening the environment (indirect entomophagy). Market perspectives for the middle of this decade indicate that most of the demand will be from the feed sector (as pet food or livestock feed production). Undoubtedly, this sector is gaining momentum and its potential relies not only in food, but also in feed in the context of a circular economy.

## 1. European Market of Insects

In the last decade, the insect sector has increased worldwide, also reaching popularity via mass media communications, mostly when new regulations or outcomes are released in relation to its potential in all areas of food chain production. Indeed, insects, when compared to conventional livestock animals, require low amounts of water, land, and feed to produce the same quantity of nutritional molecules [1]. Insects, as poikilotherm animals, can convert feed (substrates) with high efficiency in their body, decreasing the feed conversion ratio. Several international agencies and organizations have identified insects as one of the players in the commercial food chain in the future and as an active actor in reducing the environmental impact while increasing animal production [1,2]. Several international organizations are supporting the sector worldwide, such as IPIFF (International Platform of Insects as Food and Feed, Brussels, Belgium), AFFIA (Asian Food and Feed Insect Association, Bangkok, Thailand), NACIA (North America Coalition for Insect Agriculture, Chicago, IL, USA), and IPAA (Insect Protein Association of Australia, Canberra, ACT). Edible insects are becoming a part of the human diet, directly or indirectly, in many regions of the world [3,4], even if they currently represent a niche market in Western societies [5]. According to the Global Market Insights report, in 2019, the size of the edible insects market exceeded 112 million USD [6]. Though the global market data for edible insects are inconsistent, also given their short market history and consumer availability, different reports point to an overall growth of the market for edible insects [6,7,8]. The Global Market Insights report estimated that the edible insects market will grow by 47% between 2019 and 2026 [6]. It is expected that the largest increase might happen in North America and Europe [9]. The global edible insects market is segmented not only with regard to geography, but also by insect species, product type (e.g., whole insects, insect meal, and insect powder), and application (e.g., food and feed) [10]. As regards geography, the Asia–Pacific region led by Thailand, China, and Vietnam dominated the market in 2019 with around 41% of the market share in terms of revenue, followed, respectively, by Europe (22%) (led by the UK, the Netherlands, and France), Latin America (21%), North America (13%), and the Middle East and Africa (3%) [7]. Based on the insect type, beetles dominated the global edible insects market in 2019 with around 30% of the market share in terms of revenue, followed by caterpillars and Hymenoptera, respectively [7]. Insects and insect-based products are consumed in different forms: it is possible to find whole insects, or insects processed into food ingredients (e.g., flour or powder), which are then incorporated into final products (e.g., energy bars, burgers, or compound feed) [11,12]. Insects can be eaten roasted, fried, or boiled as a whole (which is the traditional way of preparation in most tropical countries), or they can be dried and ground and can then be added to foods otherwise [13].

In the past decade, in the edible insects market, numerous entrepreneurs and companies have become active in the production of insects [5]. Several initiatives have gradually transitioned from ambitious startups to well-established operators that are about to rapidly grow their production capacity. In Europe, the insect production sector was initially based mainly on small- to medium-sized startups, which undertook insect breeding in zoological gardens for biocontrol purposes or the production of animal feed [14]. Nowadays, the situation is different, and it is possible to find a certain number of insect feed business operators (iFeedBOs) (some of them are also active in food production activities) and some insect food business operators (iFoodBOs).

In the market factsheets developed each year by the IPIFF, it is possible to find specific information about these two kinds of operators. In Europe, from the 500 tons of insect-based products (whole insects, insect ingredients, and products with added edible insects) in 2019, the market will expand to 260,000 tons by 2030 [15], with powder/insect ingredients accounting for over 75% of the total. Insect FoodBOs comprise micro-companies (81%) with fewer than 10 employees, followed by small (16%) and medium-sized (3%) companies, with the number of employees between 10 and 50 or 50 and 250, respectively. The level of investment varies a lot among the operators. For most of them (63%), the investment is under 500,000 euros, and only 3% receive investments up to 25 million [16]. With the growth of the insect sector and growing investment, more jobs will be created (not only direct jobs, but also indirect ones, such as in specialized retail, logistics, administration, or research). Insect FoodBOs are involved in the different stages of insect production: primary production, processing into insect ingredients, and preparation of insect-based food. In all, 28% of the iFoodBOs are involved in all of the stages, 19% are involved in production and processing, 12%—in processing and preparation, 36%—in preparation, 3%—in production, and almost 3%—in the selling of different edible insect products to end consumers through their respective channels (e.g., online platforms). The majority of iFoodBOs are concentrated in northern European countries, led by the United Kingdom, Germany, and Belgium, and followed by the Netherlands, France, Finland, and Denmark [12]. As a matter of fact, according to some authors [1,17], the Netherlands has to be considered as the European “hub” of early research associated with the human consumption of insects in the first decade of the 21st century. The insect FoodBOs segment their final market, choosing between the national, European, and extra-European markets. In 2020, the majority (more than 60%) of the European iFoodBOs primarily focused the sale of products in the country of production. However, by 2025, iFoodBOs are expected to concentrate most of their activity on the EU market, even if they decide to maintain their activity at both the national and international level. As regards iFeedBOs, according to the IPIFF, most of them are SMEs (i.e., small enterprises with 10–50 employees or medium-sized enterprises with 50–250 employees). Micro-enterprises (1–10 employees) also represent more than 40% of the companies active in feed production. By 2030, it is expected that almost one out of two FBOs will be a large enterprise—with more than 250 employees. Insect FeedBOs have managed to raise over one billion euros in investments—figures that may reach three billion euros by 2025 [18].

By the middle of the decade, most of the demand for insect meal will likely lie in the pet food sector (almost 40–50% of the insect meal produced) and in aquaculture feed production (reaching 25–35% in terms of the share) [19]. Indeed, until 2021, in the European Union, insects as feed were only authorized for aquaculture (Commission Regulation 2017/893) and pet nutrition. Recently, the EU member states voted positively on a regulation aimed at enabling the use of insect-processed animal proteins (PAPs) in poultry and pig nutrition (Commission Regulation 2021/1372 of 17 August 2021). Thus, according to the IPIFF forecasts [18], the next relevant market for insects as iFeedBOs in terms of the quantity of insect meal sold will be the poultry (20–30%) and pig markets (5–15%), which will see a rapid increase following entry into force of the approval of insect PAPs.

The potential use of edible insects in European countries is currently debated among many stakeholders of the food supply chain. This paper aims to provide a concise but comprehensive state of the art on how insects could be used as food in light of the new legislative framework and recent EU novel food authorizations. In addition, we underline the crucial role of consumer acceptance of edible insects and reflect on how consumers will play a role in this sector. Our discussion takes into consideration the current situation at the European level in the framework of research policy and business perspective.

## 2. Regulation of Insects as Food in the EU

Before insect-based food products started to attract a significant level of attention in the European food market, small-scale production and trade of edible insects had not been considered sufficiently important to be subject to legislative matters or safety standards supervision [20]. In fact, in most European countries, the consumption of edible insects is still very low and often seen as socially improper [21]. This is also because in Europe, edible insects and insect-based products are classified as novel foods (NFs) [11], that is, food products that do not have a history of human consumption within the region or country in question or, more specifically, any food that was not consumed “significantly” prior to May 1997, according to the EU regulations. However, interest in insect-based products has been increasing in the last few years, triggered especially by the potential environmental, economic, and food security benefits that insects could offer [22,23]. The novelty of using insects for human consumption, as well as the high interest from the public and the media in such products [5], poses important questions and concerns regarding the risks derived from insect production, processing, and consumption [24]. Moreover, the need in reliable guidelines has concerned producers and slowed down the growth of the sector, as also highlighted jointly in 2018 by NACIA, IPIFF, AFFIA, and IPAA [5]. Answering these questions, and therefore ensuring the safety of consumers, requires valid scientific assessments that adequately inform policymakers with the responsibility to authorize the incorporation of such products into the market.

**Figure 1:**
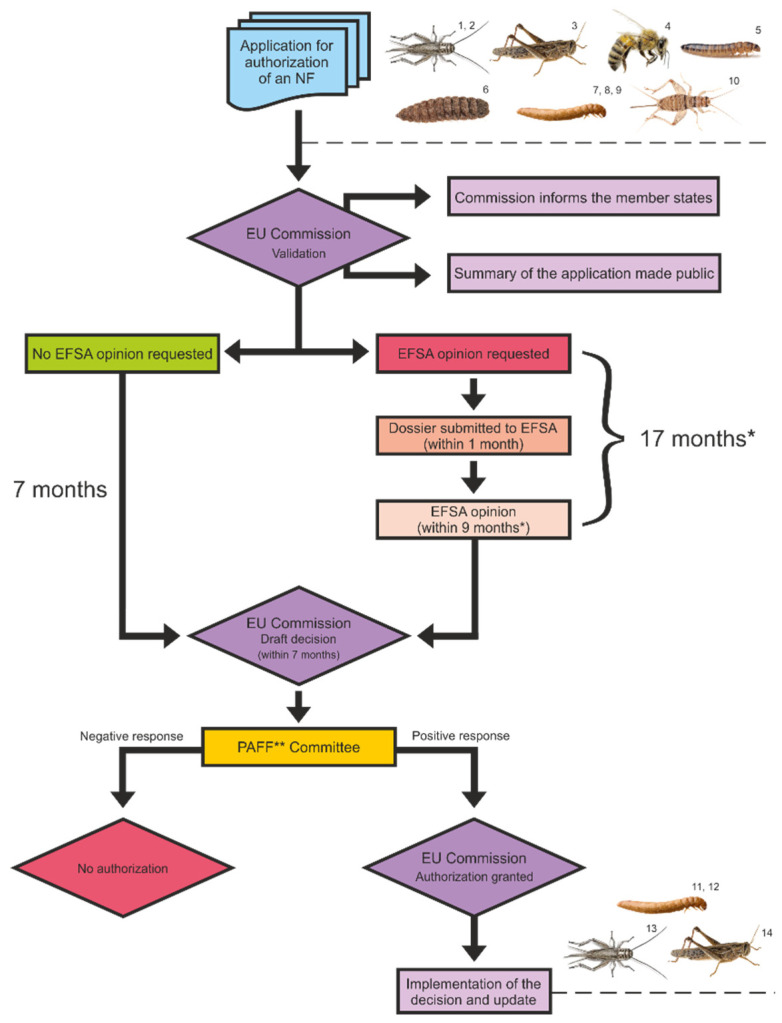
Novel food (NF) authorization process. * This amount of time can be longer if the EFSA requests additional information from the applicant. ** The Standing Committee on Plants, Animals, Food and Feed. ^1,2^ *Acheta domesticus* (Belgian Insect Industry Federation—Belgium; Cricket One—Vietnam). ^3^ *Locusta migratoria* (Belgian Insect Industry Federation—Belgium). ^4^ *Apis mellifera* (Finnish Beekeepers’ Association—Finland). ^5^ *Alphitobius diaperinus* (Proti-Farm Holding NV—the Netherlands). ^6^ *Hermetia illucens* (Enorm Biofactory A/S—Denmark). ^7,8,9^ *Tenebrio molitor* (Nutri’Earth—France; Belgian Insect Industry Federation—Belgium; Ynsect—France). ^10^ *Gryllodes sigillatus* (SAS EAP Group—Saint-Orens-de-Gameville, France). ^11,12^ *Tenebrio molitor* (SAS EAP Group—France; Protix Company—the Netherlands). ^13^ *Acheta domesticus* (Protix Company, Fair Insects BV—Dongen, the Netherlands). ^14^ *Locusta migratoria* (Protix Company, Fair Insects BV—Dongen, the Netherlands).

Consequently, in 2015, the European Food Safety Authority (EFSA) published its first scientific opinion on the risks associated with the production and consumption of farmed insects as food and feed [25]. It covered considerations about potential allergenic and environmental risks, as well as chemical and biological hazards linked to external factors such as the methods used for their production, the substrates they are fed on, and the lifecycle stage at which they are harvested, among others. The EFSA concluded that as long as insects are fed with currently permitted feed materials, the potential occurrence of such hazards is expected to be similar to that of other non-processed protein sources. This means that insects can only be safely reared on substrates of vegetable origin or specific allowed animal-origin materials, preventing the possibility of using substrates containing manure and other waste materials. This opinion laid the groundwork for a new European regulation on insects as NF, i.e., Regulation (EU) 2015/2283. According to this new regulation, insect food products may be commercialized only if authorized by the European Commission (EC). The process of obtaining such authorization is very straightforward: anyone who intends to place an NF on the EU market must submit an application to the EC. After the application is verified and validated, it becomes available to all the member states, and the EFSA is requested to provide a scientific safety assessment within nine months from a valid application. Based on this opinion, authorization is granted or not. This regulation also indicates the specific insects that can be utilized as NFs, labeling recommendations for insect-based products, as well as general conditions for the inclusion of NFs in the union list, which is updated by the EC and serves as a reference for economic operators who wish to place an authorized NF on the market (Figure 1).

More recently, Regulations (EU) 2017/2469 and (EU) 2017/893 specify some rules regarding the administrative and scientific requirements to an application to request authorization of commercialization of an NF in the EU market, as well as some amendments of the general criteria for insect and insect protein production, with particular attention to the substrate options that can be used to rear them, which are limited to those permitted for other livestock species.

Although, on the one hand, the highly restrictive regulations for the production and commercialization of insects and insect-based products in Europe allow governments to ensure the safety of consumers, on the other hand, they also slow down the development of an industry capable of offering potential environmental and economic benefits [26]. At the same time, in some countries, the lack of formal standards for production have led to uncertainty and concern among many insect farmers, providing an unstable basis for the industry (though also opening the possibility for the industry itself to innovate in the development of standards) [27]. Therefore, new scientific evidence enabling an optimized and improved assessment and the identification of specific critical control points in the entire production and processing chain of insects is essential.

## 3. European Authorizations

Based on the regulation of NFs, as of December 2021, four authorizations deal with insects. First, the EC authorized the placing on the EU market of dried *Tenebrio molitor* larvae (yellow mealworm) as an NF. The application requested for dried *Tenebrio molitor* larvae to be used as whole, dried insects in the form of snacks and as a food ingredient in several food products, the target population being the general population. The applicant (SAS EAP Group, France) made a request to the Commission for the protection of the proprietary data submitted in the application.

Based on the applicant’s request, whole, dried larvae or larval powder were proposed to be used as ingredients in food products, defined, using the FoodEx2 hierarchy, as snacks other than chips and similar (maximum use level, ML = 100 g NF/100 g product), protein and protein components for sports people (ML = 10 g NF/100 g), biscuits (ML = 10 g NF/100 g), legumes-based dishes (ML = 10 g NF/100 g), and uncooked pasta-based dishes (ML = 10 g NF/100 g) (see Table 1 for a complete list of products) [28].

The Commission also authorized the placing on the market of a second insect, *Locusta migratoria* (migratory locust), as an NF on 12 November 2021. The request dealt with frozen and dried forms of the insect to be used in different food categories (Table 1). The applicant was Fair Insects BV (Protix Company, the Netherlands). The NF consists of frozen, dried, and powder forms of the migratory locust, intended to be marketed as a snack or as a food ingredient in several food products. Moreover, Protix Company requested data protection [29]. On 8 December 2021, two requests by Fair Insects BV (Protix Company, the Netherlands) were authorized as NFs. The authorized requests dealt with frozen and dried formulations of whole *Tenebrio molitor* and frozen and dried formulations of whole *Acheta domesticus* (house cricket) (Table 1). The applicant requested protection of the data for both NFs [30,31].

The other 10 applications dealt with *Tenebrio molitor* (Nutri’Earth—France; Belgian Insect Industry Federation—Belgium; Ynsect—France), *Acheta domesticus* (Belgian Insect Industry Federation—Belgium; Cricket One—Vietnam), *Gryllodes sigillatus* (SAS EAP Group—France), *Locusta migratoria* (Belgian Insect Industry Federation—Belgium), *Alphitobius diaperinus* (Proti-Farm Holding NV—the Netherlands), *Hermetia illucens* (Enorm Biofactory A/S—Denmark), and *Apis mellifera* (Finnish Beekeepers’ Association—Finland).

Based on the applicants’ proposed uses, all of the NFs were listed in the “Snacks other than chips and similar” products as whole products. Thus, all of the enterprises will be able to place on the market products containing only insects that have been dried, frozen, or ground into powder (in relation to the request). These include bakery products, such as bread, biscuits, and crackers containing mealworms and crickets. Six different types of products referred to pasta-like products, all listed in the two requests made by Fair Insects BV (Protix Company) for mealworms and crickets. Moreover, three different types of pizza were listed for these requests.

Edible insects are commonly presented and positioned to consumers as meat alternatives [17,32]. This argument is not supported by research or entrepreneurial ideas, but is wrongly based on the idea that insects could be a substitute for meat products, providing significant environmental benefits. Edible insects are more than an alternative to meats; especially in relation to the nutritional–economic status of the consumers/country, insects could be positioned as a major protein or energy food, decreasing the gaps between the rich and poor nutritional diets across the developing and the developed countries (without negatively affecting the environment). Furthermore, in the developed countries, consumers are unwilling to accept the direct substitution of a “nice” slice of meat with a “strange” dish of insects (food neophobia) [33,34,35,36]. In this contest, meat-like products are only partially reported in the lists. Indeed, sausages, meatballs, and meat burgers were listed with an inclusion of approximately 30–40% (*w*/*w*) of frozen locusts, mealworms, and crickets, or approximately 10–16% (*w*/*w*) of dried/powdered insects. Contrarily, meat imitates will be intended as more insect-based, with a maximum of 50% of dried/powdered insects or 80% of frozen insects.

Interestingly, a high number of vegetable-based foods was reported in the requests. Vegetable-based dishes, meals, soups, salads, and canned/jarred items were requested by all the applicants. Moreover, oilseed and primary derivatives from nuts and similar seeds were listed in the applications with quite high maximum contents of insects (respectively, 20–30 dried or powder NF g/100 g product and 40 frozen NF g/100 g products).

It seems like producers are going to use edible insects in beverages more as a curiosity, and likewise the caterpillar of *Comadia redtenbacheri* (gusanos rojos in Spanish) in a mezcal traditional recipe [37]. The percentages in beer and beer-like, mixed alcohol drinks, as well as unsweetened spirits and liqueurs, range between 1% and 2% *w*/*w*.

Foods intended for sports people are also listed as “protein and protein components for sports people” and whey powder. In these products, the insect contents are 10%, 20%, or 40%.

## 4. Consumer Acceptance of Insects as Food

In the past few years, a considerable number of studies has been published on consumers’ acceptance of alternative proteins, including edible insects as food [32]. Several recent up-to-date reviews on eating insects and insect-based foods provide a critical overview within the EU [4], as well as globally [38]. In addition, Sogari et al. [39] provided a comprehensive perspective on the overall state of research activity on consumer attitude and behavior toward entomophagy without date restrictions. Their results showed that the number of publications has increased substantially since 2015, after the publication of the FAO report “Edible Insects: Future Prospects for Food and Feed Security” [1,5], and the trend has maintained since then [38]. Most of these studies focused on European consumers, with only a few of them including a cross-country comparison (e.g., Italian and Dutch samples in [40]). As a result of the majority of these studies being conducted in Western countries, researchers have investigated consumers’ reactions to processed insect-based foods such as snacks (e.g., biscuits, chips, and bars) rather than whole and traditional dishes with insects [4,41].

This rising publication trend calls out for a need to provide a thematic synthesis. First, compared to even few years ago, as Dagevos [38] suggested, we have gained a considerable understanding of consumers’ acceptance of insect-eating. It is now clear that a high level of food neophobia (i.e., fear of new foods) implies a low inclination to consider insects as a food to try and purchase, regardless of the respondents’ origin [42,43]. Another motivation of Western consumers’ aversion to eating insects is disgust, a primary emotion and a major aversive reaction toward insect consumption since research on entomophagy started [5,41].

The aversion to insects as a food is also strictly linked to the food culture [24]. Several studies have shown that the stronger the gastronomic culture within the society, the greater the rejection, and vice versa [4,40]. This is also linked to other people’s opinions (social norms), which could strongly affect the acceptance of consuming this novel food. On the contrary, individuals who have already had a positive experience of eating edible insects show a higher willingness to eat insects in the future [4]. Moreover, previous experience of eating insects plays a significant role in the willingness to repeat consumption due to a positive perception of tastiness (e.g., expected liking) and reduced food neophobia [38,41,44].

Several studies have analyzed the effects of information about the benefits of entomophagy on the attitudes toward insect food products, indicating that this strategy increased insects’ acceptance [32,41]. For example, Mancini et al. [4] showed how an education lecture about the ecological, safety, nutritional, and taste-related aspects of insect-eating increased the willingness to try insects and insect-containing foods among a group of university students. However, consumers’ response to these information treatments is strongly correlated with subjective interests in the nutritional or environmental benefits of the eaten food [38]. Thus, it is likely that young people, such as students, who are sensitive to the current challenges in food sustainability [45] will more likely be responsive to information about the health or sustainability aspects of insects as food than older consumers. In fact, early adopters of insect food products are often identified in young adults [4], especially males [32,46]. In particular, it has been noted by Jones [47] that in her study, many young people of school age welcomed the opportunity to have their preconceptions surrounding what is acceptable challenged in light of a more sustainable food system. Besides gender and age, the dietary regimen also plays a role with respect to consumers’ attitudes toward entomophagy. For example, Elorinne et al. [46] showed that non-vegan vegetarians’ attitudes toward eating insects were the most positive, whereas vegans held the most negative attitude, considering eating insects as immoral and irresponsible. In fact, in the Netherlands, supermarkets display insect-based convenience foods such as burgers and nuggets in the same section as vegetarian products [48].

Familiar food preparation is another driver for insect consumption [4,44]. Thus, the current market strategy is to develop highly processed insect-based foods that are familiar in Western diets such as burgers, bread, biscuits, crackers, crisps, candy bars, shakes, soups, sauces, and pasta [12,38]. This rationality of “hiding” the appearance of insects and including them in crushed, bruised, or powdered forms, rather than visible and whole, is supported by a large amount of empirical evidence [4,41]. It seems obvious that to reduce disgust-based aversion and consequently raise the acceptance toward entomophagy, insects need to be processed in a form that resembles known products (i.e., familiar-looking).

Another recent stream of consumer acceptance studies focuses on using insects as a feed source, which is sometimes defined as “indirect entomophagy” [49]. From the literature, it seems that consumers will have higher acceptance of using insects as feed for farmed animals than as food for human consumption [32]. In fact, in a recent study, Menozzi et al. [50] found that there is a potential consumer interest in considering insects as a protein substitute in the poultry sector.

## 5. Conclusions and Future Prospects

World population growth and the increase in food demands push scholars to research alternative protein sources for both human and animal consumption. As presented in the previous sections, the last decade has seen a growing interest from the public and private sectors in research in the sphere of edible insects, as well as significant steps forward from the legislative perspective in the EU. Moving from entomophobic countries such as most of those in Europe to more open entomophilic societies will be favored by common and shared stakeholder efforts, including national governments, the research community, and the private sector [24]. This latter will include actors across the production, supply, and consumption of food, which will be crucial to industry success [48]. Undoubtedly, this sector is gaining momentum, and its potential relies not only on food and feed, but also on the context of a circular economy. In particular, insects have the potential to convert a wide range of organic byproducts into food and feedstuffs, which then go back into the production cycle [51]. However, due to the current lack of evidence related to the safety of the final products, EU regulations still prohibit the use of waste products as a substrate for growing edible insects. Our review showed how the most edible insect species, i.e., crickets, mealworms, grasshoppers, and locusts, investigated in consumer studies [41] are those already authorized by the EFSA (i.e., *Tenebrio molitor* and *Locusta migratoria*), or currently subject to a safety evaluation by the authority. As suggested by House [17], these species are the “industry standard” food insects in Europe as the positive result of a number of technical, practical, and legislative factors.

Although some adventurous consumers and sensation-seekers could try a visible and whole insect [38], it seems clear that the marketing strategy adopted by most insect companies is to develop processed products (e.g., bakery, meat, pasta, and pizza products) in which insects are “hidden” in the form of a powder or similar. In addition, it has to be highlighted how even if the current legislation and industry are contributing to the idea that insects are a legitimate and edible (i.e., safe) food, this does not mean that they will instantly be accepted and consumed [48]. Acceptance will especially depend upon education, especially in the context of schools and young individuals, with the aim of changing mis- and preconceptions about edible insects [47].

The prospect for future research should then concentrate on different aspects: (i) The economic convenience of introducing insect meals into animals’ diets, both in the developing and the developed countries. Future studies on insects as feed and food should better investigate the structure cost and profitability of insect farms. This type of analysis could facilitate the development of other farms to undertake the production of insects and also support the sustainable development of this sector from the circular economy perspective. (ii) Consumers’ willingness to pay for eating insects, insect-based foods, and animal feed with insects as meal. Thus far, a considerable number of consumer-oriented studies have focused on consumers’ attitudes and behavior toward entomophagy. However, studies investigating consumers’ willingness to pay for such products in real settings are lacking. Future research on this aspect is also important to push farms to embrace this new sector of activity. (iii) The role of governments in supporting the insect farming sector. Governments play a decisive role in facilitating a shift toward new and sustainable food solutions. The sustainability of food systems is a global issue, and food systems will have to adapt to face diverse challenges. As mentioned in the “Farm to Fork strategy,” all actors of the food chain must play their part in achieving the sustainability of the food chain. Farmers and producers need to transform their production methods more quickly and make the best use of nature-based, technological, digital, and space-based solutions to deliver better climate and environmental results, increase climate resilience, and reduce and optimize the use of inputs. However, these solutions require not only human and financial investments, but also a collective approach involving public authorities at all levels of governance that could increase participation and provide a voice for farms and producers in global processes related to food. (iv) Promoting the use of insects from the packaging and labeling standpoint. Attractive naming and descriptions, communication of health benefits, branding (logo), and product image are important to consumers’ perceptions of insect foods. Future entomophagy research should therefore focus on improving marketing strategies to ensure that insects and insect-based products become more appealing. A correct communication strategy could ameliorate access to insects and reduce future generations’ unfamiliarity, inappropriateness, and disgust that are the major causes of the non-acceptance of insects today.

## Figures and Tables

**Table 1:** Food products and the maximum use levels reported in the four EFSA opinions on insects as novel foods (NFs).

	Maximum Use Level (g NF/100 g)								
Food Category	*T. molitor* ^1^	*L. migratoria* ^2^			*T. molitor* ^2^			*A. domesticus* ^2^	
	W/D/P	F	D	P	D	P	F	D/P	F
Beans and vegetable meal								5	15
Beer and beer-like beverages		2	2	2	1	1	1	1	1
Biscuits	10								
Biscuits, sweet, plain					8	8	30	8	30
Bread and rolls with special ingredients added								10	30
Caesar salad		15	5	5				5	15
Canned or jarred legumes		20	15	15					
Canned/jarred vegetables		20	15	15					
Cereal bars					15	15	30	15	30
Chickpeas (without pods)		40	20	20	30	30	40	25	40
Chips/crisps					20	20	40		
Chocolate and similar		30	10	10	10	10	30	10	30
Crackers and breadsticks					10	10	30	10	30
Dried pasta					5	5	15	1	3
Dried stuffed pasta					15	15	30	15	30
Frozen yoghurt		15	5	5	5	5	15	5	15
Hummus								5	15
Legume (beans) soup		15	5	5				5	15
Legume-based dishes	10	15	5	5	5	5	15		
Meatballs					16	16	40	16	40
Meat burgers (no sandwiches)					16	16	40	16	40
Meat imitates		80	50	50	50	50	80	50	80
Mixed alcoholic drinks		2	2	2	1	1	1	1	1
Mixed vegetable soup		15	5	5					
Mixed vegetable soup (dry)		15	5	5				5	15
Multigrain bread and rolls					10	10	30		
Mushroom soup		15	5	5				5	15
Mushroom soup (dry)		15	5	5				5	15
Oilseeds		40	20	20	30	30	40	25	40
Onion soup		15	5	5				5	15
Pasta-based dishes, cooked					5	5	15		
Pasta, filled, cooked								5	15
Pasta-based dishes, uncooked	10								
Pizza and pizza-like dishes		15	5	5	5	5	15		
Pizza and similar with cheese and vegetables								5	15
Pizza and similar with cheese, vegetables, and fruits								5	15
Potato soup		15	5	5				5	15
Potato-based dishes		15	5	5	5	5	15		
Potatoes and vegetable meal								5	15
Pre-mixes (dry) for baked products					15	15	30	15	30
Prepared pasta salad		15	5	5				5	15
Primary derivatives from nuts and similar seeds		40	20	20	30	30	40	25	40
Protein and protein components for sports people	10								
Sausages		30	10	10					
Snacks other than chips and similar	100	100	100	100	100	100	100	100	100
Soups and salads					5	5	20		
Tartar sauce					10	10	30	15	30
Tomato soup		15	5	5				5	15
Tomato soup (dry)		20	5	5				5	20
Tortilla chips								20	40
Tree nuts		40	20	20	30	30	40	25	40
Unsweetened spirits and liqueurs		2	2	2	1	1	1	1	1
Whey powder					20	20	40	20	40

W, whole; F, frozen; D, dried; P, powder. Applicant: ^1^ SAS EAP Group [28]; ^2^ Fair Insects BV (Protix Company) [29,30,31].
